# Muscular Rheumatism

**Published:** 1894-11-10

**Authors:** E. Mansel Sympson

**Affiliations:** Surgeon to the Lincoln County Hospital


### MUSCULAR RHEUMATISM.

By E. Mansel Sympson, M.A., M.D.Cantab., M.R.C.S., Surgeon to the Lincoln County Hospital.

Muscular rheumatism can generally be easily and clearly distinguished from the other complaints to which the name of rheumatism is applied. Acute rheumatism, or rheumatic fever, with its high fever, swelling and painfulness of joints; chronic rheumatism, where the pain and swelling of joints are present without the fever; rheumatic arthritis, with the peculiar distortion of joints, and an accompanying train of nervous symptoms, are clinically, and, I believe, pathologically distinct from true muscular rheumatism. Except in the form of "stiff-neck" (which is common in children), this is a disease of middle life, and selects very frequently vigorous persons, and especially men. It figures under other names according to the region of the body attacked, as lumbago or sciatica, &c. Pain in the muscles of the shoulder, which is not uncommon, has received no special name

The immediate causes of muscular rheumatism are exposure to cold, damp, and sudden muscular exertion (an occasional one in this list being a round at golf). The common stiff-neck, from a draught of cold air, is a good example. And muscular exertion frequently occasions an apparent recrudescence of the complaint.

*Muscular* rheumatism assumes apparently that the seat of the disease is in the muscles. With regard to one part of it, *i.e.*, sciatica, it seems fairly certain that this is essentially a neuritis, an inflammation of the sciatic nerve, from the pain and tenderness along its course, and when it is put on stretch, as well as from the paresis or actual paralysis, and wasting of muscles, that sometimes comes on as the result. This, then, has no existence in the muscles. Again, in the ordinary forms of lumbago, there is pain chiefly or solely when there is movement of the muscles of the back, but there is no tenderness to touch, unless the muscle happens to be stimulated to contract. In this form it seems likely to be more of a neuritis, an irritation or inflammation of the nerve endings in muscle, and so strictly comparable to that already mentioned in sciatica, which is so often present, more or less actively, in the subjects of lumbago. If this be so, the complaint has evidently little relationship to acute and chronic rheumatism, and only resembles osteo-arthritis in being a degeneration. The comparatively poor results obtained by the use of the salicylates tends to favour this view. Dr. Haig, indeed, is inclined to regard lumbago as the result of uric acid attacking the planes of fibrous tissue between and encompassing the muscles

The treatment of muscular rheumatism by alkalies is fairly satisfactory, though very depressing to the patients, and the disease *per se* is sufficiently depressing. I have tried giving acids by themselves, without much effect, but I am quite certain that arsenic has a powerful effect for good in this complaint. It seems to take away the pain sooner than anything else (except, of course, opium, but it returns with tolerable certainty), and, although several patients have been under observation for years, they do not seem to suffer afterwards from relapses or fresh attacks, so much as patients who have had other drugs, which rather tends to support a suggestion of Mr. Jonathan Hutchinson, that arsenic is a preventive against "chills," and patients who have taken it for various reasons have spontaneously told me that they were not so subject to "taking cold," although they had resumed their ordinary work. Its mode of action in lumbago, probably, from what has been said above, is that of a nerve tonic. I find 3—5 minims of either liq. arsenicalis or liq. arsenici hydrochlor. three or four times a day, quite sufficient, and I combine the latter with ten to fifteen minims of ac. nitro hydrochlor. dil. and some bitter infusion, such as gentian. A free action of the bowels is most important in treating muscular rheumatism, and it is perhaps best attained by suitable doses of Hunyadi Janos early in the morning. A little wine, such as Burgundy or hock, is valuable when taken with meals, which should be light but nutritious, plenty of fish, and eggs and milk, but little or no butchers' meat.

Rest in bed, if it can be obtained, is a great step of itself to cure. Local measures may be mentioned in the order in which their usefulness has struck the writer. Hot water bottles applied to the affected part, hot bran bags, poultices, mustard and linseed, mustard leaves, various liniments (aconite, belladonna, and camphor). Not infrequently massage has disappointed me. Dry cupping I have seen used with excellent results, though I have not tried it myself.

